# Inactivation of 3-hydroxybutyrate dehydrogenase type 2 promotes proliferation and metastasis of nasopharyngeal carcinoma by iron retention

**DOI:** 10.1038/s41416-019-0638-8

**Published:** 2019-12-10

**Authors:** Bo Li, Zhipeng Liao, Yingxi Mo, Weilin Zhao, Xiaohui Zhou, Xiling Xiao, Wanmeng Cui, Guofei Feng, Suhua Zhong, Yushan Liang, Chunping Du, Guangwu Huang, Ping Li, Xue Xiao, Xiaoying Zhou, Rensheng Wang, Zhe Zhang

**Affiliations:** 1 Department of Radiotherapy, First Affiliated Hospital of Guangxi Medical University, Nanning, China; 2 Department of Otolaryngology-Head & Neck Surgery, First Affiliated Hospital of Guangxi Medical University, Nanning, China; 3 Department of Research, Affiliated Tumor Hospital of Guangxi Medical University, Nanning, China; 4 Life Science Institute, Guangxi Medical University, Nanning, China; 5 Department of Pathology, First Affiliated Hospital of Guangxi Medical University, Nanning, China; 6 Department of Pathology, Affiliated Tumor Hospital of Guangxi Medical University, Nanning, China

**Keywords:** Head and neck cancer, Tumour-suppressor proteins, Head and neck cancer, Tumour-suppressor proteins, Head and neck cancer

## Abstract

**Background:**

3-Hydroxybutyrate dehydrogenase type 2 (BDH2) is known to catalyse a rate-limiting step in the biogenesis of the mammalian siderophore and regulate intracellular iron metabolism. Here we aim to explore the expression and possible function of BDH2 in nasopharyngeal carcinoma (NPC).

**Methods:**

The transcription and protein expression of BDH2 in NPC were determined by both real-time RT-PCR and immunohistochemistry staining assays. Cell proliferation, migration and invasion were evaluated by MTT assay, wound-healing assay and Transwell assay, respectively. The profile of genes regulated by restoring BDH2 expression in NPC cells was analysed by cDNA microarray. The level of iron in NPC cells was detected by iron colorimetric assay.

**Results:**

The expression of BDH2 was significantly downregulated in NPC. Ectopic expression of BDH2 inhibited NPC cell proliferation and colony formation. Meanwhile, BDH2 suppressed the migration and invasion of NPC cells by reversing the epithelial–mesenchymal transition (EMT). In addition, a higher level of BDH2 decreased the growth and metastasis of NPC cells via reducing intracellular iron level.

**Conclusions:**

Our findings suggest that BDH2 may be a candidate tumour-suppressor gene in NPC. Decreasing intracellular iron could be an effective therapeutic approach for NPC.

## Background

Nasopharyngeal carcinoma (NPC) is a malignant tumour with the distinct geographical, racial and ethnic distribution.^1^ For most western countries, NPC is an uncommon malignancy with an incidence of <1/100,000 people.^2^ However, this cancer is prevalent in southern China, where it is the third most common malignancy among males, with an incidence of 15–30/100,000 people.^3^ Three major aetiological factors are involved in the carcinogenesis of NPC, including genetic susceptibility, environmental factors and Epstein–Barr virus (EBV) infection.^1,3^ Currently, the major therapeutic options of NPC include radiotherapy, chemotherapy and chemoradiotherapy. Despite significant progress in intensity-modulated radiation therapy and chemoradiotherapy, therapy for NPC still remains a great challenge because of radio- and chemo-resistance, disease relapse and distant metastasis. Therefore, we need novel and effective therapeutic strategies for NPC.^4^

Iron-generated reactive oxygen/nitrogen species and aldehydes lead to genetic alteration, and elevated iron levels might increase the risk of cancer.^5^ Excess iron levels have been found carcinogenic in many animal models.^6^ Iron acts as a cofactor for many enzymes including ribonucleotide reductase, an enzyme involved in NDA synthesis. To sustain proliferation, cancer cells need to increase the iron assimilation and consequently modulate the expression of proteins involved in iron uptake. Decreased expression of the iron exporter ferroportin leading to iron retention was found to be associated with poor prognosis in breast cancer.^7^

3-Hydroxybutyrate dehydrogenase type 2 (BDH2), also called DHRS6 and located on the human chromosome 4q24, encodes a member of the short-chain dehydrogenase/reductase family.^8^ As a key enzyme in ketogenesis, BDH2 mediates the first step of ketone body degradation from 3-hydroxybutyrate to acetoacetate in the liver.^9^ In tissues other than the liver, BDH2 has a physiological role in the utilisation of cytosolic ketone bodies, as a secondary system for energy supply in starvation or to generate precursors for lipid and sterol synthesis.^8^ BDH2 also manipulates intracellular iron homoeostasis by containing an iron-responsive element and mediating cellular iron tracking by catalysing the synthesis of the mammalian siderophore that binds labile iron.^10^

The roles of BDH2 differ in several human malignancies. BDH2 mRNA expression was higher in the bone marrow of patients with acute myeloid leukaemia (AML). Overall survival was shorter in AML patients with higher BDH2 expression, and the response to intensive induction chemotherapy was lower. BDH2 works as an anti-apoptotic factor, mediated by survivin via a caspase-3- independent pathway.^11^ Besides, BDH2 was found upregulated and correlated with tumour location or TNM stage in oesophageal cancer tissue. Knockdown of BDH2 induced cell apoptosis via a caspase-3-dependent apoptosis pathway.^12^ On the other hand, the expression of BDH2 was decreased in hypoxic glioma cells.^13^ However, the expression and function of BDH2 remain unclear in NPC.

Here, we explored the expression of BDH2 in NPC, addressed its effect on malignant behaviour and possible molecular mechanisms involved.

## Methods

### Cell lines and culture conditions

The immortalised human nasopharyngeal epithelial cell lines NP69 and NP460 were maintained in keratinocyte/serum-free medium (Invitrogen, Carlsbad, CA, USA).^14,15^ The NPC cell lines C666-1, HONE1, CNE1, HK1, 5–8 F, 6–10B and TW03 were maintained in high glucose DMEM (Invitrogen) supplemented with 10% foetal bovine serum (Invitrogen), 100 U/mL penicillin and 100 μg/mL streptomycin. Cells were incubated at 37 °C in a humidified chamber containing 5% CO_2_.^16–19^

Primary NPC tumour biopsies were obtained from 43 newly diagnosed and untreated cases from donors, with informed consent, in the Department of Otolaryngology, Head and Neck Surgery, First Affiliated Hospital of Guangxi Medical University (Nanning, China). The diagnoses were established by experienced pathologists according to the World Health Organization (WHO) classification. Forty normal nasopharyngeal epithelium (NNE) tissue samples were collected as controls. In total, 21 NNE and 23 NPC biopsies were used for RNA extraction, and 19 NNE and 20 NPC biopsies were fixed in 10% paraformaldehyde embedded in paraffin.

### Antibodies, plasmids and transfection

We used the following antibodies: BDH2 (1:1000, HPA004428, Sigma-Aldrich, St. Louis, MO, USA), β-catenin (1:1000, sc-376841, Santa Cruz Biotechnology, Santa Cruz, CA, USA), E-cadherin (1:1000, #3195P) and vimentin (VIM; 1:1000, #5741P, both Cell Signaling Technology, Beverly, USA), secreted protein acidic and cysteine rich (SPARC; 1:1000, #66426-1, SANYING, Wuhan, China) and GAPDH (1:10000, #5174P, Cell Signaling Technology). The secondary antibody 680RD goat anti-mouse and IRDye 800CW goat anti-rabbit antibodies were from LI-COR Biosciences (Lincoln, NE, USA).

BDH2 plasmid was purchased from Origene (Rockville, MD, USA); the open-reading frame of BDH2 was subcloned into the pCMV6-Entry vector. The protocol of transfection has been described.^20^

### RNA extraction, cDNA synthesis and real-time PCR

The RNA extraction, cDNA synthesis and real-time PCR were performed as previously described.^20^ The primer sequences were as follows: BDH2-F 5′-GCTTCCAGCGTCAAAGGAGTT-3′, BDH2-R 5′-CAGTTGCGAATCTTCCCGTC-3′, GAPDH-F 5′-GCTCAGACACCATGGGGAAG-3′, GAPDH-R 5′-TGTAGTTGAGGTCAATGAAGGGG-3′, epithelial cell adhesion molecule (EPCAM)-F 5′-CTGGCCGTAAACTGCTTTGT-3′, EPCAM-R 5′-AGCCCATCATTGTTCTGGAG-3′, SPARC-F 5′-GTGCAGAGGAAACCGAAGAG-3′, SPARC-R 5′-TCATTGCTGCACACCTTCTC-3′, cadherin 1 (CDH1)-F 5′-GTCAGGTGCCTGAGAACGAG-3′, CDH1-R 5′-GCCATCGTTGTTCACTGGAT-3′, VIM-F 5′- GAACTTTGCCGTTGAAGCTG-3′, VIM-R 5′-TCTCAATGTCAAGGGCCATC-3′, matrix metalloproteinase 2 (MMP2)-F 5′-GCCTCCTCCTGACATTGACCT-3′ and MMP2-R 5′-AACACAGCCTTCTCCTCCTG-3′.

### Immunohistochemistry staining

Tissues were cut into 3-μm-thick sections and incubated for 1 h with 3% hydrogen peroxide to eliminate endogenous peroxidase activity after deparaffinisation and rehydration. After antigen retrieval, sections were incubated with BDH2 antibody (1:1000, HPA004428, Sigma, St. Louis, MO, USA) at 4 °C overnight, then secondary antibody (ZB-2305, ZSGB-BIO, Beijing) for 1 h at room temperature. A 3,3′-diaminobenzidine (DAB) reagent (ZLI-9018, ZSGB-BIO, Beijing) was used for peroxidase reaction, and haematoxylin for counterstaining. Images were acquired under a microscope (C-5050, Olympus, Japan). The results were independently evaluated in a blinded manner by two pathologists. The intensity of BDH2 staining was scored as described.^20^

### Cell proliferation assay

The cell proliferative capacity was measured by MTT assay as previously described.^20^

### Acetoacetate colorimetric detection assay

Acetoacetate Colorimetric Assay Kit (Biovision, Milpitas, CA, USA) was used to determine intracellular acetoacetate content, following the manufacturer’s instructions.

### Colony-formation assay

Cells were seeded in six-well plates at 2 × 10^2^ cells per well. After 14 days, Giemsa-stained colonies formed were photographed and counted by using Quantity One v4.4.0 (Bio-Rad, USA).

### In vivo tumorigenicity assay

Five 5-week-old male BALB/c-nu nude mice (Vital River Laboratory Animal Technology, China) were injected with 1.0 × 10^6^ pCMV6-Entry-5–8F cells and an equal number of BDH2–5–8F cells into the left flank by subcutaneous injection. All mice were raised by professional breeders from Laboratory Animal Center of Guangxi Medical University in a barrier system with certain temperature and humidity in the SPF animal lab and were randomly divided into two groups. The tumour volume was assessed by 2D measurements at 0, 3, 5, 8, 11 and 14 days. Tumour volume was calculated as volume (mm^3^) = length × width^2^ × 0.5. Then mice were killed by cervical dislocation and tumours were removed. All animal experimental procedures were performed under protocol No. 201808022, approved and regulated by The Animal Care & Welfare Committee of Guangxi Medical University. All methods were carried out in accordance with the Guiding Opinions on the Treatment of Laboratory Animals issued by the Ministry of Science and Technology of the People’s Republic of China and the Laboratory Animal-Guideline for Ethical Review of Animal Welfare issued by the National Standard GB/T35892-2018 of the People’s Republic of China.

### cDNA microarray analysis and bioinformatics analysis

Affymetrix GeneChip Genome U133 Plus 2.0 expression array and Gene-Cloud of Biotechnology Information (GCBI) platform (www.gcbi.com.cn) were used to analyse genes with differential expression between BDH2–5–8F and pCMV6-Entry-5–8F cells. The microarray data are available via the following accession identifier on the NCBI-GEO database: GSE119642. The expression level of BDH2 in TCGA data was performed by using GEPIA (http://gepia.cancer-pku.cn).

### Wound-healing assay

Cells (8 × 10^5^ per well) were seeded into 12-well plates and allowed to adhere overnight with DMEM medium without serum. Monolayer cells were scratched by using the ibidi Culture-Insert (No. 80209, ibidi, Germany). Images were acquired under a light microscope (TS100, Nikon, Japan) two times (0 and 18 h), and analysed by using Image J v1.51k (NIH, USA).

### Transwell assay

Cells (2.5 × 10^4^ per well) were seeded in the upper chamber of 24-well Bio-Coat Invasion Chambers (BD, USA) coated with Matrigel. The lower chamber was filled with DMEM medium with 10% FBS. Non-invading cells were removed by using a cotton-tipped swab at 48 h. Migratory and invasive cells on the lower membrane surface were fixed with 1% paraformaldehyde, stained with 0.5% crystal violet and photographed.

### Western-blot analysis

Equal amounts of protein were separated by electrophoresis on 4–12% SDS-PAGE and transferred to the nitrocellulose membrane (Thermo Fisher Scientific, USA), which was blocked in 5% bovine serum albumin (BSA) in 1 × Tris-buffered saline containing 0.1% Tween 20. The membrane was incubated with primary antibodies at 4 °C overnight followed by the appropriate peroxidase-conjugated secondary antibodies at room temperature for 2 h. LI-COR Odyssey (Lincoln, NE, USA) was used to detect fluorescent signals.

### Iron colorimetric detection assay

Iron Colorimetric Assay Kit (Biovision, Milpitas, USA) was used to determine intracellular iron content, following the manufacturer’s instructions.

### Statistical analysis

All data were analysed by using SPSS 17.0 (SPSS Inc., Chicago, IL, USA). Data are expressed as mean ± SD. The statistical analysis was performed by Independent-Sample Test. Statistical significance was considered at *p* < 0.05.

## Results

### BDH2 is downregulated in NPC cell lines and tissue

**Fig. 1:**
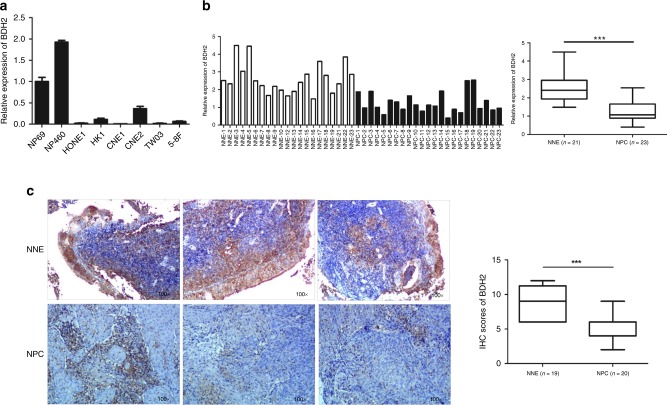
Downregulated mRNA and protein level of BDH2 in NPC. **a** Relative BDH2 transcriptional level was determined by real-time RT-PCR in six NPC cells and two noncancerous nasopharyngeal epithelial (NNE; NP69, NP460) cell lines by using the 2^−ΔCt^ method and normalised to GAPDH. **b** Real-time RT-PCR analysis of relative BDH2 mRNA expression in normal (*n* = 21) and tumour (*n* = 23) tissues. **c** Representative images of immunostaining for BDH2 expression in three NNE and three NPC samples (magnification × 100). Horizontal line indicates the median, box edges are 25–75 percentiles and whiskers are 10 and 90 percentiles. ***p < 0.001

To reveal the potential role for BDH2 in NPC, we examined the mRNA expression of BDH2 in NPC primary tumours and NPC cell lines by real-time RT-PCR. BDH2 level was decreased in six NPC cell lines (HONE1, HK1, CNE1, CNE2, TW03 and 5–8F) as compared with two noncancerous nasopharyngeal epithelial cell lines (NP69 and NP460) (Fig. 1a). As well, BDH2 expression was lower in NPC tissues than NNE tissues (Fig. 1b). We also evaluated BDH2 expression in patient-derived biopsies by immunohistochemistry staining (IHC). BDH2 protein was detected in the cytoplasm of both NNE and NPC cells. Notably, BDH2 expression was significantly lower in NPC than NNE (Fig. 1c).

To confirm our findings, we analysed RNA-sequencing data from the cancer genome atlas (TCGA) and found BDH2 transcription downregulated in several kinds of cancer, including cervical and endocervical cancers (CESC), cholangiocarcinoma (CHOL), prostate adenocarcinoma (PRAD) and lung squamous cell carcinoma (LUSC) (Supplementary Fig. 1), suggesting that inactivation of BDH2 may be a common event in human cancers.

### Ectopic expression of BDH2 inhibits the proliferation and colony formation of NPC cells

**Fig. 2:**
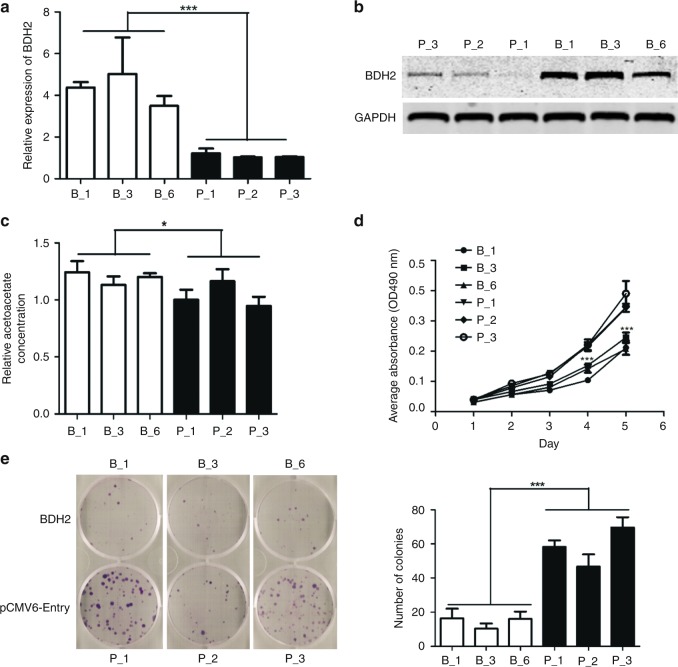
Ectopic expression of BDH2 inhibits the proliferation and colony formation of NPC cells. **a**, **b** The expression of BDH2 in 5–8F cells stably transfected with BDH2-PCMV6-Entry (B_) and control vector (P_), respectively, confirmed by both real-time RT-PCR and western-blot analysis. **c** Acetoacetate detection assay in BDH2–5–8F and pCMV6–5–8F cells. **d** Growth curve of three sub-colonies of BDH2–5–8F and pCMV6–5–8F cells determined by MTT assay. **e** Colony formation of BDH2–5–8F and pCMV6–5–8F cells. Data are mean ± SD. * *P* < 0.05, ***P < 0.001

To access the function of BDH2, NPC cell line 5–8F was stably transfected with control vector and BDH2 expression plasmid, respectively. We had three single clones of pCMV6-Entry-5–8F (p_1, p_2 and p_3) and BDH2–5–8F (b_1, b_3 and b_6), respectively. The expression of BDH2 was confirmed by both real-time RT-PCR and western-blot assay (Fig. 2a, b). To further confirm the function of BDH2, we detected an increase in intracellular acetoacetate level in BDH2–5–8F cells (Fig. 2c). In addition, BDH2–5–8F cells grew more slowly than control cells (Fig. 2d), with reduced capacity for colony formation (Fig. 2e).

### BDH2 suppresses NPC tumour growth in vivo

**Fig. 3:**
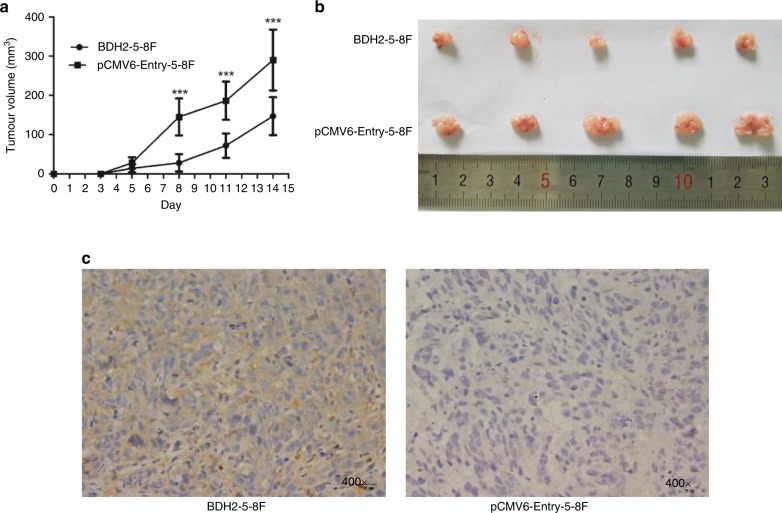
BDH2 suppresses NPC tumour growth in vivo. **a** The volume of tumours derived from BDH2–5–8F-1 and pCMV6–5–8F-1 cell injection in mice was measured at days 0, 3, 5, 8, 11 and 14 after inoculation. **b** Xenograft tumours were removed at 14 days after inoculation in nude mice (*n* = 5). Data are mean ± SD. **c** Immunohistochemical staining of BDH2 in removed tumours (magnification × 400). ***P < 0.001

To determine whether the effect of BDH2 expression on tumour suppression could be reproduced in vivo, we injected BDH2–5–8F and pCMV6-Entry–5–8F cells in the left flank of nude mice. Fourteen days later, mice were killed, and tumour volume was smaller with inoculation of BDH2–5–8F than pCMV6-Entry–5–8F cells (Fig. 3a, b). IHC of tumours confirmed the increased expression of BDH2 in tumours from BDH2–5–8F cell lines (Fig. 3c).

### BDH2 reverses the epithelial–mesenchymal transition (EMT) in NPC cells

**Fig. 4:**
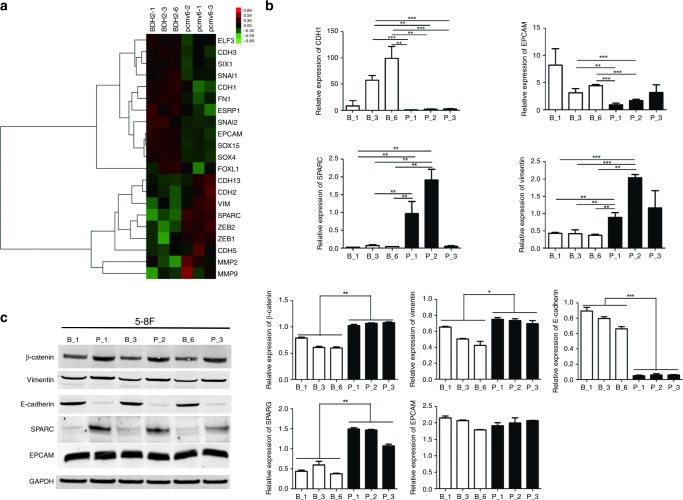
BDH2 reverses the epithelial–mesenchymal transition (EMT) in NPC cells. **a** Microarray assay: heatmap showing expression of 21 genes involved in EMT in BDH2-overexpressing 5–8F cells as compared with pCMV6-Entry–5–8F cells. **b** Relative expression of CDH1, EPCAM, SPARC and VIM analysed by real-time RT-PCR in BDH2–5–8F and pCMV6-Entry–5–8F cells. **c** Western-blot analysis of protein expression of E-cadherin, β-catenin, vimentin, SPARC and EPCAM in cells. GAPDH was used as an endogenous control. Data are mean ± SD (*n* = 3). *P < 0.05; **P < 0.01; ***P < 0.001

**Fig. 5:**
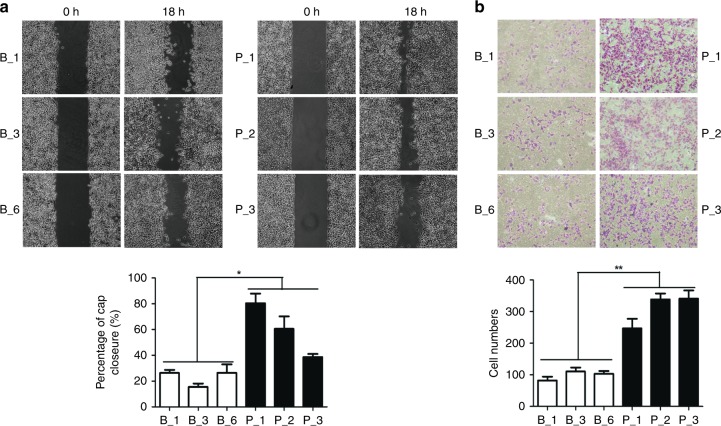
Ectopic expression of BDH2 inhibits migration and invasion of NPC cells. **a** Migration of three sub-colonies of BDH2–5–8F and pCMV6–5–8F cells examined by wound-healing assay at 0 and 18 h. The percentage of wound width for each sample was calculated. Magnification × 100. **b** Transwell assay of invasive capacity of BDH2–5–8F and pCMV6–5–8F cells. The blue dots represent the invading cells stained with crystal violet. The number of invading cells was counted. Data are mean ± SD (*n* = 3). *P < 0.05, **P < 0.01

By microarray analysis, we found that the top two genes with differential expression, EPCAM (~5-fold) and SPARC (~18-fold), involved in EMT, were altered by overexpressing BDH2 in 5–8F cells (Supplementary Table 1). Therefore, we analysed 21 EMT markers (Fig. 4a). The typical EMT marker, CDH1 (upregulated ~3-fold) was influenced by BDH2 expression. As well, the transcription levels of EPCAM and CDH1 were upregulated in BDH2–5–8F versus PCMV6-Entry–5–8F cells, and those of SPARC, VIM and MMP2 were downregulated (Fig. 4b). In addition, 5–8F cells with overexpressed BDH2 showed significantly increased protein expression of E-cadherin, and reduced expression of β-catenin, SPARC and VIM (Fig. 5c), while the expression of EPCAM was not affected by BDH2. These data suggest that the expression level of BDH2 might contribute to the migration and invasion of NPC cells.

### Ectopic expression of BDH2 inhibits migration and invasion of NPC cells

To investigate the effect of BDH2 on the motility and invasive capacities of NPC cells, we performed wound-healing assay and Transwell assay, respectively. We observed that the gap closure is slower in BDH2–5–8F cells (Fig. 5a), and fewer BDH2–5–8F cells invaded the extracellular matrix gel in contrast with control cells (Fig. 5b), suggesting that higher expression of BDH2 attenuates the metastasis of NPC cells by reversing EMT.

### BDH2 inhibits proliferation by reducing intracellular iron content

**Fig. 6:**
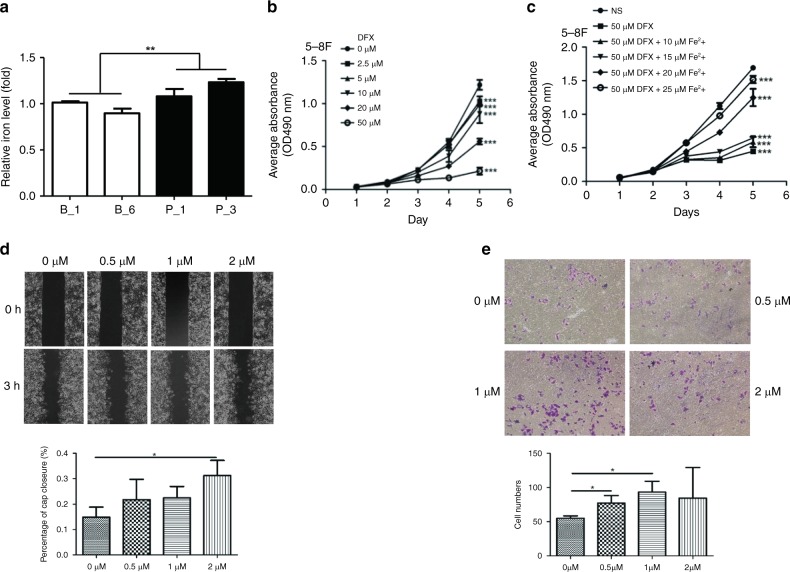
Iron retention accelerates cell proliferation, migration and invasion of NPC cells. **a** Relative concentration of intracellular iron calculated as the total amount of intracellular iron per microgram protein in two sub-colonies each of BDH2–5–8F and pCMV6-Entry–5–8F cells. **b** MTT assay of proliferation of 5–8 F cells under deferasirox (DFX) treatment. **c** Proliferation of cells after adding iron (II) sulfate heptahydrate to saturate the iron-chelating ability of deferasirox (50 μM). Data are mean ± SD (*n* = 5). **d** Wound-healing assay was performed in 5–8 F cells treated with iron (II) sulfate heptahydrate at 0, 0.5, 1 and 2 μM, respectively. The wound width was measured at 0 and 3 h. Magnification × 100. **e** Invasive capacity of 5–8F cells, with iron supplement, was assessed by Transwell assay. The number of invading cells was counted. Data are mean ± SD (*n* = 3). *P < 0.05; **P < 0.01; ***P < 0.001

BDH2 deficiency has been shown to promote iron overload.^21^ To confirm the ability of BDH2 to regulate iron levels in NPC cells, we detected intracellular iron deposits by iron colorimetric assay. The iron level was lower with two single clones of BDH2–5–8F cells than two clones of pCMV6-Entry–5–8F cells (Fig. 6a), which suggests that downregulation of BDH2 causes iron retention in NPC cells. Re-expression of BDH2 may decrease intracellular iron content, thereby inhibiting the proliferation of NPC cells.

### Elevated iron amount increases proliferation, migration and invasion of NPC cells

To further evaluate the effect of iron on growth, we treated 5–8F and HK1 cells with different concentrations of deferasirox, an iron inhibitor. Deferasirox decreased the proliferation of these two cell lines; the inhibition effect was more obvious with increased deferasirox concentration (Fig. 6b; Supplementary Fig. 1A). With iron supplemented in increasing concentrations, the capacity for cell proliferation was restored gradually (Fig. 6c; Supplementary Fig. 2B). Thus, intracellular iron level may be positively associated with the growth ability of NPC cells. High intracellular iron level has been verified to contribute to metastasis progression.^22^ EGFR also plays a role in regulating iron homoeostasis to increase iron import for cancer cell.^23^ The wound-healing assay and Transwell assay were performed to determine the effect of the iron supplement on the motility and invasive capacities of NPC cells. The data showed that iron supplement significantly increased the migration of 5–8F cells at concentration 2 μM (Fig. 6d). In addition, 0.5 and 1 μM iron supplement significantly promoted invasion (Fig. 6e). We demonstrated that iron supplement increases migration and invasion of NPC cells. Moreover, as shown in Supplementary Fig. 3, the migration of HK1 cells transiently expressed BDH2 faster after iron supplementation. This evidence supports that the motility of NPC cells impeded by overexpressing BDH2 relies on the iron decline.

## Discussion

Here, we report for the first time that BDH2 is inactivated in NPC. We also found that the transcription of BDH2 was downregulated in a variety of tumours, indicating that the lower expression of BDH2 is universal in malignancies. To date, what causes BDH2 inactivation in tumour has not been addressed yet. It has been reported that BDH2 expression is downregulated during inflammatory and ER stress response in macrophages.^24^ One could speculate that the inflammatory microenvironment of tumour cells could negatively influence BDH2 expression activity. In contrast, the expression of BDH2 was reported to be aberrantly high in patients with acute myeloid leukaemia and oesophageal carcinoma,^12^ which imply a tumour-specific effect of BDH2 expression.

In this study, we have investigated the role of BDH2 in the pathogenesis of NPC. Ectopic expression of BDH2 in NPC cells reduced cell proliferation, colony formation and suppressed tumorigenesis in the in vivo model. The restored expression of BDH2 suppressed migration and invasion of NPC cells by reversing the EMT process. Altogether, our data support a role of BDH2 as a tumour-suppressor gene in NPC.

Proper energy metabolism is an essential factor in the survival and progression of normal cells through a cell cycle. Metabolic alterations have been observed in various malignancies and considered as a hallmark of cancer.^25^ Metabolic reprogramming of cancer cells provides additional energy to support their malignant behaviour, characterised by accelerated proliferation, resistance to apoptosis, evasion of immune surveillance and maintenance of the status of cancer stem cells.^26–28^ Previously, we reported that accumulating lipid droplets (LDs) in NPC cells is positively correlated with their malignant behaviour, indicating that LDs might be an energy fuel of NPC.^29^ In several types of cancer, obesity and excess accumulation of adipose tissues are widely considered as high-risk factors.^30^ In addition, lipid accumulation significantly reduces the drug efficacy of chemotherapy and anti-angiogenic drug in cancer.^31,32^ Targeting lipid metabolism combined with other treatment may be a novel strategy for cancer patients with multidrug resistance or obesity.

In organs other than the liver, BDH2 dehydrates β-hydroxybutyric acid to form one of the endogenous ketone body molecules, acetoacetate,^9^ considered to be a nutritional source for tumours carrying the V600E mutant form of BRAF protooncogene.^33,34^ Interestingly, ketone bodies also reduce the growth of pancreatic cancer and cause apoptosis.^35^ We found that restoring the expression of BDH2 in NPC cells increased the intracellular acetoacetate level. Early, we showed that the use of exogenous acetoacetate does not affect the migration of NPC cells,^20^ which suggests that the tumour-suppressor function of BDH2 still does not rely on acetoacetate production. Intriguingly, a recent study found that acetoacetate produced by overexpressing BDH2 in liver cancer cells inactivates the macrophage migratory inhibitory factor (MIF), therefore impeding the recruitment of tumour-associated macrophage (TAM), an important target for cancer treatment.^36,37^ We speculated that acetoacetate affects cancer cells through the paracrine mode of action, other than autocrine. More experiments are necessary to address this interesting issue in the future.

Iron metabolism is a chain of chemical reactions that supports iron homoeostasis at both the systemic and cellular levels. Iron is directly related to cell proliferation and growth. It can generate reactive oxygen species (ROS) by participating in the Fenton reaction that produces hydroxyl radicals. ROS can damage DNA and be mutagenic. Thus, iron is fundamental to cell survival and may also be associated with carcinogenesis.^38^

In recent years, numerous disorders, such as cancer and neurodegenerative diseases, have been linked to deregulated iron homoeostasis.^33,34^ Cellular iron metabolism of iron includes three major processes: iron uptake, storage and export of iron. Iron carrier proteins, such as transferrin receptors in glioblastoma and ferritin in serum, were upregulated, thereby increasing iron uptake.^35,36^ The decrease in expression of an iron exporter protein ferroportin is associated with a poor prognosis for breast cancer.^39^ In addition, accumulation of iron supports cancer stem cells and induces drug resistance, transforming the cells into a malignant phenotype and leading to treatment failure.^35,37^ In patients with NPC, higher serum ferritin levels are closely associated with the development of metastasis.^40^ Conversely, high expression of the ferritin heavy chain in NPC cell lines significantly reduces the ability of these cells to proliferate.^41^ Iron metabolism is not fully understood in NPC yet.

BDH2 mediates the formation of 2,5-dihydroxybenzoic acid (2,5-DHBA), a siderophore. Siderophores are small high-affinity iron-chelating compounds that function in transporting iron across cell membranes. BDH2 plays a crucial role in intracellular homoeostasis of iron and regulates the immune function of macrophages.^42,43^ Siderophore transports ferrous iron to mitochondria, since its absence leads to iron deficiency in mitochondria, and as a result, to an increase in the level of iron in cells.^10^ Mitochondria are the main place of iron utilisation. The imported iron is used to synthesise iron–sulfur clusters, which are important for the activity of the citric acid cycle enzymes, oxidative phosphorylation and mitochondrial respiration.^44^ The lack of siderophore contributes to the abnormal accumulation of intracellular iron and increases cell proliferation.^10^ Our results indicate that inactivation of BDH2 leads to iron retention in nasopharyngeal epithelial cells, so it may be important for carcinogenesis and development of NPC.

Approaches targeting cellular iron levels and iron-mediated signalling to inhibit tumour growth have been developed and applied in cancer therapy. The use of iron chelators can cause cell cycle arrest, which will subsequently lead to apoptosis, which suggests the use of iron chelators as potential anticancer agents.^45–48^ Artemisinin is toxic only to cancer cells that contain more iron and kills cancer cells by increasing the level of ROS.^49^ ROS are required to cause DNA damage during radiotherapy, the main strategy for NPC treatment. Our study showed for the first time that an iron chelator, desferrioxamine, suppresses the proliferation of NPC cells in vitro, so iron chelators can be useful for treating NPCs by suppressing cell growth and proliferation or participating in redox reactions as a radiation therapy enhancer. Thus, iron metabolism management may be a new therapeutic target in the treatment of NPC.

Thus, we found that BDH2 expression is downregulated in NPC. Overexpression of BDH2 in NPC cells decreased the levels of intracellular iron, thereby inhibiting cell proliferation. In addition, overexpression of BDH2 reduced invasion and migration of NPC cells by reversing EMT. Our results revealed a new mechanism for the carcinogenesis of NPC in that iron metabolism may influence the development of NPC. Thus, iron metabolism management can be a potential approach to prevention and treatment in NPC.

## Supplementary information

Supplementary one file

## Data Availability

The microarray data have been deposited to the NCBI-GEO database (https://www.ncbi.nlm.nih.gov/geo/) with the data set identifier GSE119642.
