# Supplementary material for: Inactivation of 3-hydroxybutyrate dehydrogenase type 2 promotes proliferation and metastasis of nasopharyngeal carcinoma by iron retention
Source: Br J Cancer. 2019 Dec 10;122(1):102–10. doi: 10.1038/s41416-019-0638-8 (PMC6964698; doi:10.1038/s41416-019-0638-8)

**Supplementary Table 1. Differential expressed genes between BDH2-5-8F and pCMV6-Entry-5-8F cells involved in EMT.**

| **Gene Symbol** | **Fold Change** | **p-value** | **q-value** | **Gene Feature** |
| --- | --- | --- | --- | --- |
| EPCAM | 5.05094 | 5.10E-05 | 0 | up |
| SPARC | -18.368 | 5.60E-05 | 0 | down |
| ESRP1 | 4.24332 | 0.000129 | 0 | up |
| FN1 | 4.20453 | 0.000134 | 0 | up |
| CDH1 | 3.45676 | 0.000159 | 0 | up |
| SOX15 | 2.2917 | 0.000412 | 0.017544 | up |
| SOX4 | 1.6781 | 0.000671 | 0.017544 | up |
| SNAI2 | 2.45743 | 0.001359 | 0.065421 | up |
| ZEB2 | -2.3556 | 0.00152 | 0.191532 | down |
| SNAI1 | 1.85111 | 0.008161 | 0.25 | up |
| CDH3 | 1.59851 | 0.01082 | 0.25 | up |
| ELF3 | 2.21585 | 0.015992 | 0.31917 | up |
| SIX1 | 1.53377 | 0.029011 | 0.367264 | up |
| ZEB1 | -1.4656 | 0.074805 | 0.956953 | down |
| MMP2 | -1.2156 | 0.107985 | 0.956953 | down |
| FOXL1 | 1.1077 | 0.18197 | 0.625508 | up |
| MMP9 | -1.1755 | 0.239068 | 0.956953 | down |
| VIM | -1.3092 | 0.283905 | 0.956953 | down |
| CDH2 | -1.3851 | 0.329143 | 0.956953 | down |
| CDH13 | -1.4293 | 0.434247 | 0.956953 | down |
| CDH5 | -1.1407 | 0.669193 | 0.956953 | down |


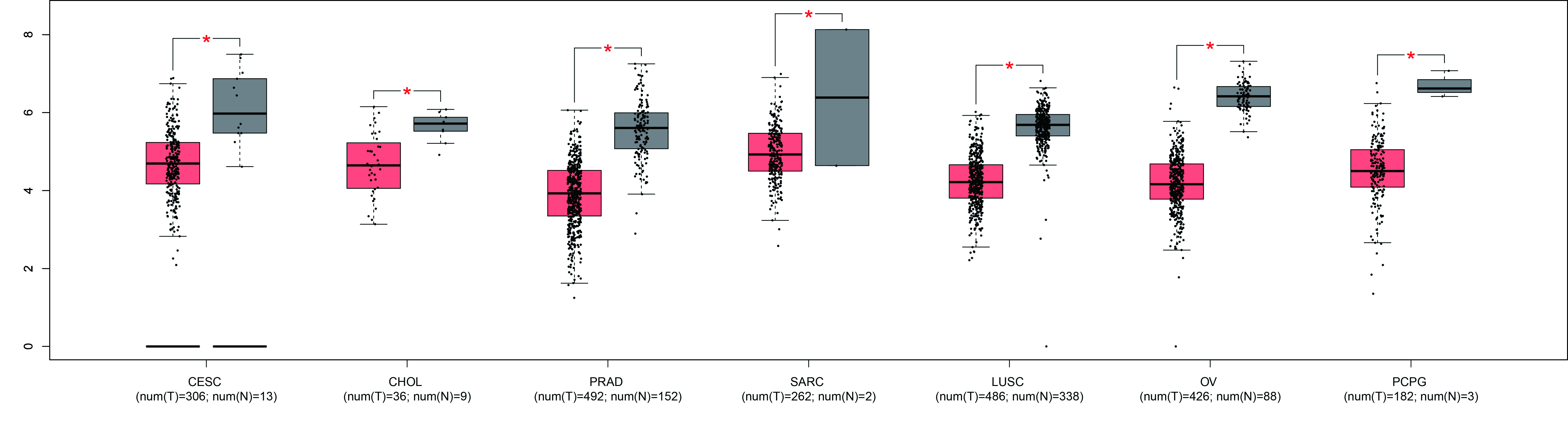


**Supplementary figure legend 1:**

The transcriptional level of BDH2 was downregulated in Cervical and endocervical cancers (CESC), Cholangiocarcinoma (CHOL), Prostate adenocarcinoma (PRAD) and Lung squamous cell carcinoma (LUSC). Based on the RNA-sequencing data from the cancer genome atlas (TCGA), the mRNA expression of BDH2 was analyzed using [a Gene Expression Profiling Interactive Analysis](http://www.baidu.com/link?url=3jlUyASVx2DXYRIkqXUMzuQDwOUKYaqGY7NH9hFM1c9ycfh3tRI6ulOP8BW_p6bV) (GEPIA) platform (<http://gepia.cancer-pku.cn/>). * P<0.05


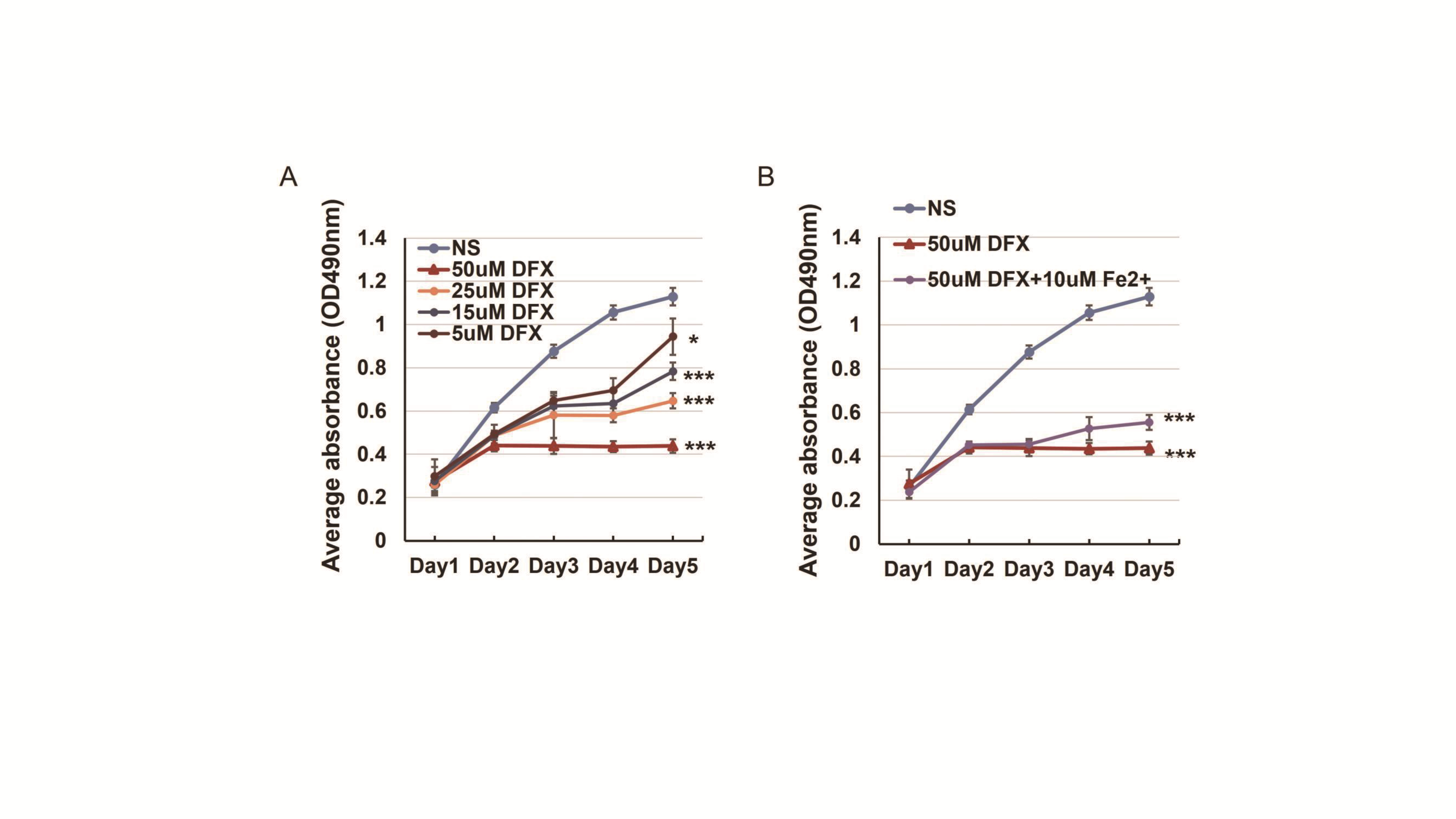


**Supplementary figure legend 2:**

Iron supplement accelerates the proliferation of HK1 cells. A: Proliferation of HK1 cells under deferasirox (DFX) treatment was assessed by MTT assay. B: Proliferation of HK1 cells after adding iron (II) sulfate heptahydrate to saturate the iron-chelating ability of deferasirox (50 μM). Data are mean ± SD (n=3). * P <0.05; ** P < 0.01; *** P < 0.001.


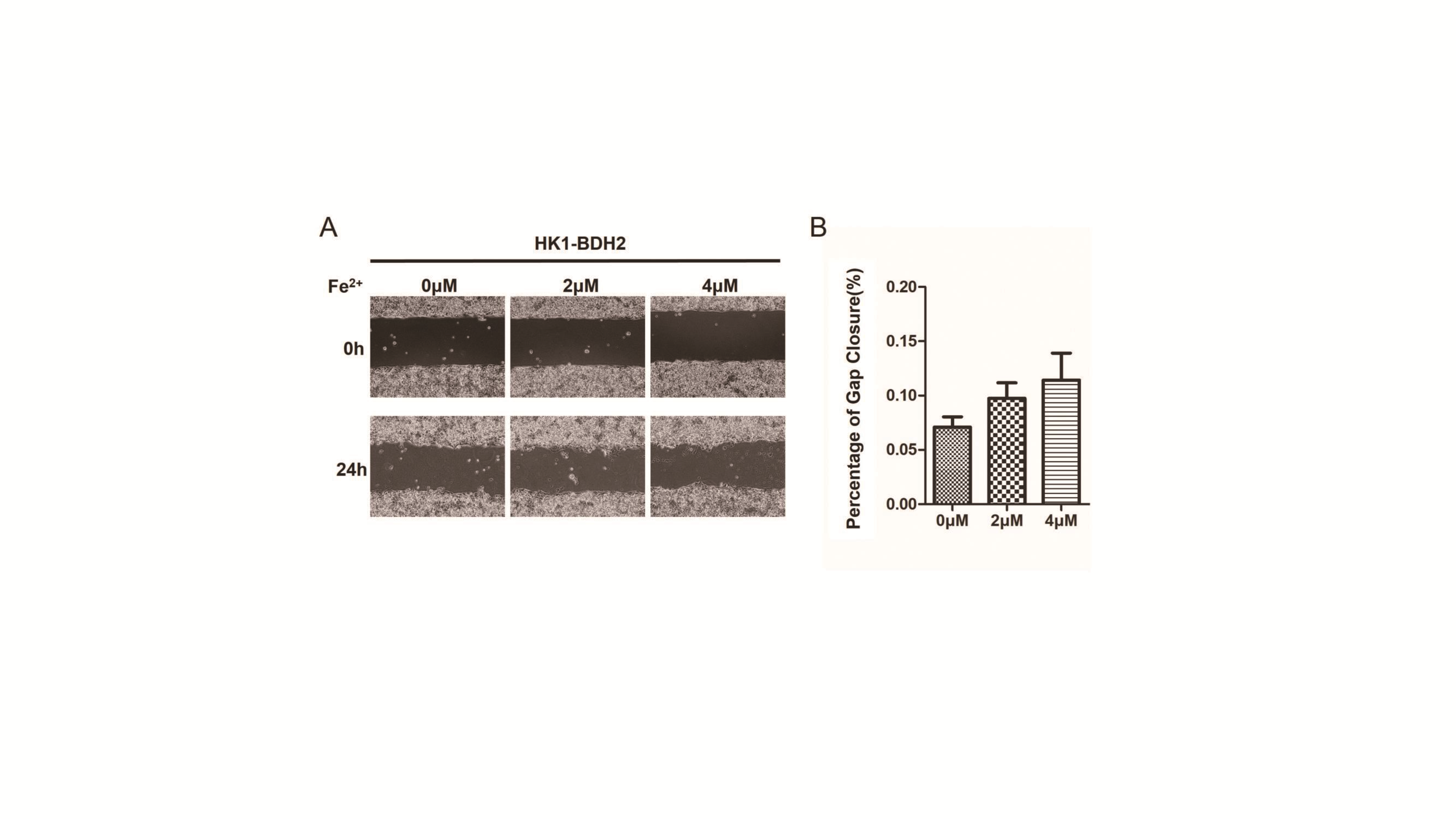


**Suppl****ementary figure legend 3:**

Iron supplement accelerates the migration of HK1 cells transiently expressed BDH2. Wound-healing assay was performed in HK1-BDH2 cells treated with iron (II) sulfate heptahydrate at 0μM, 2μM, 4μM. The wound width was measured at 0 and 24 h. Magnification ×100. Data are mean ± SD (n=3). * P <0.05; ** P < 0.01; *** P < 0.001.

**NC3RS checklist：**


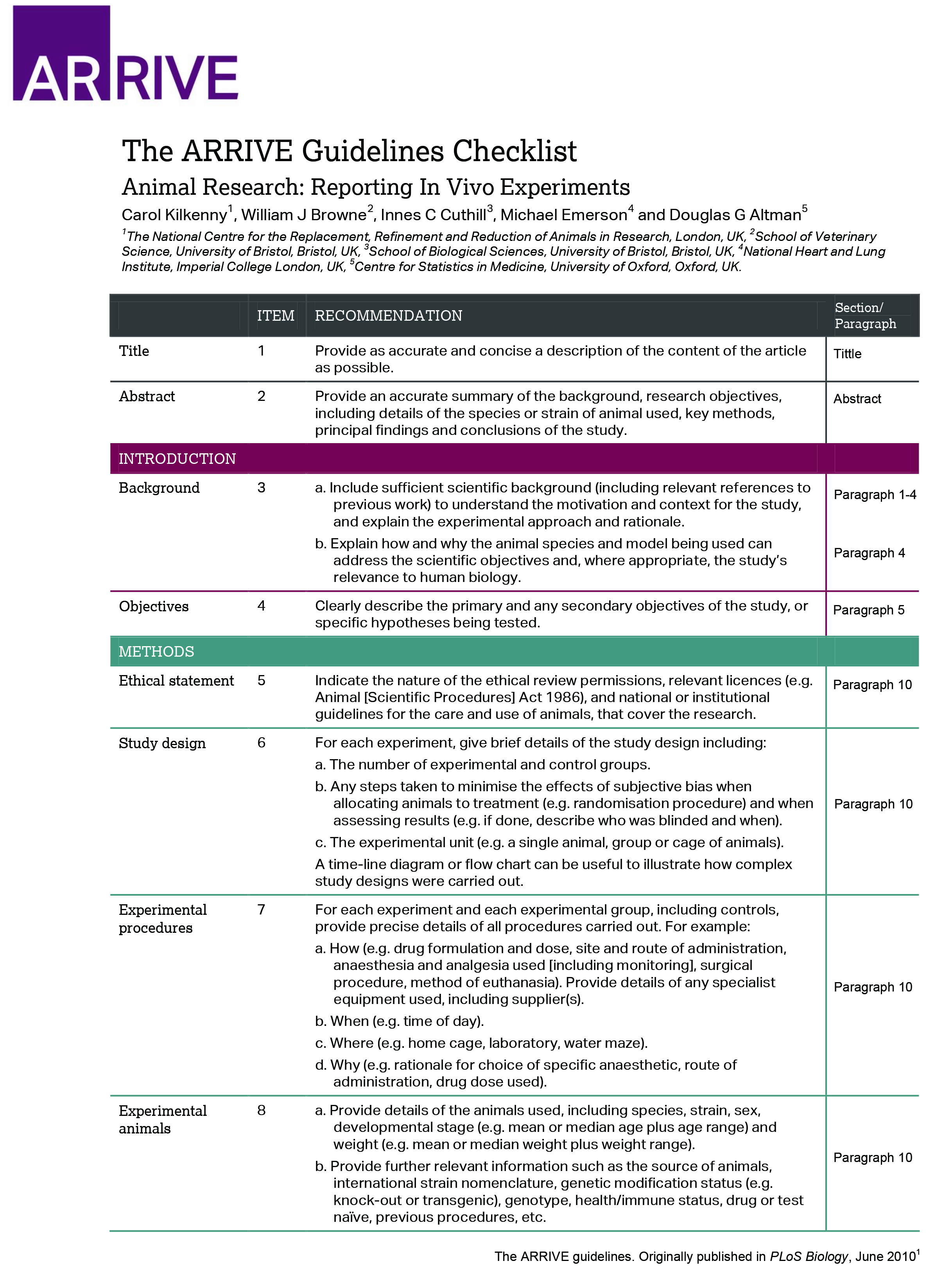


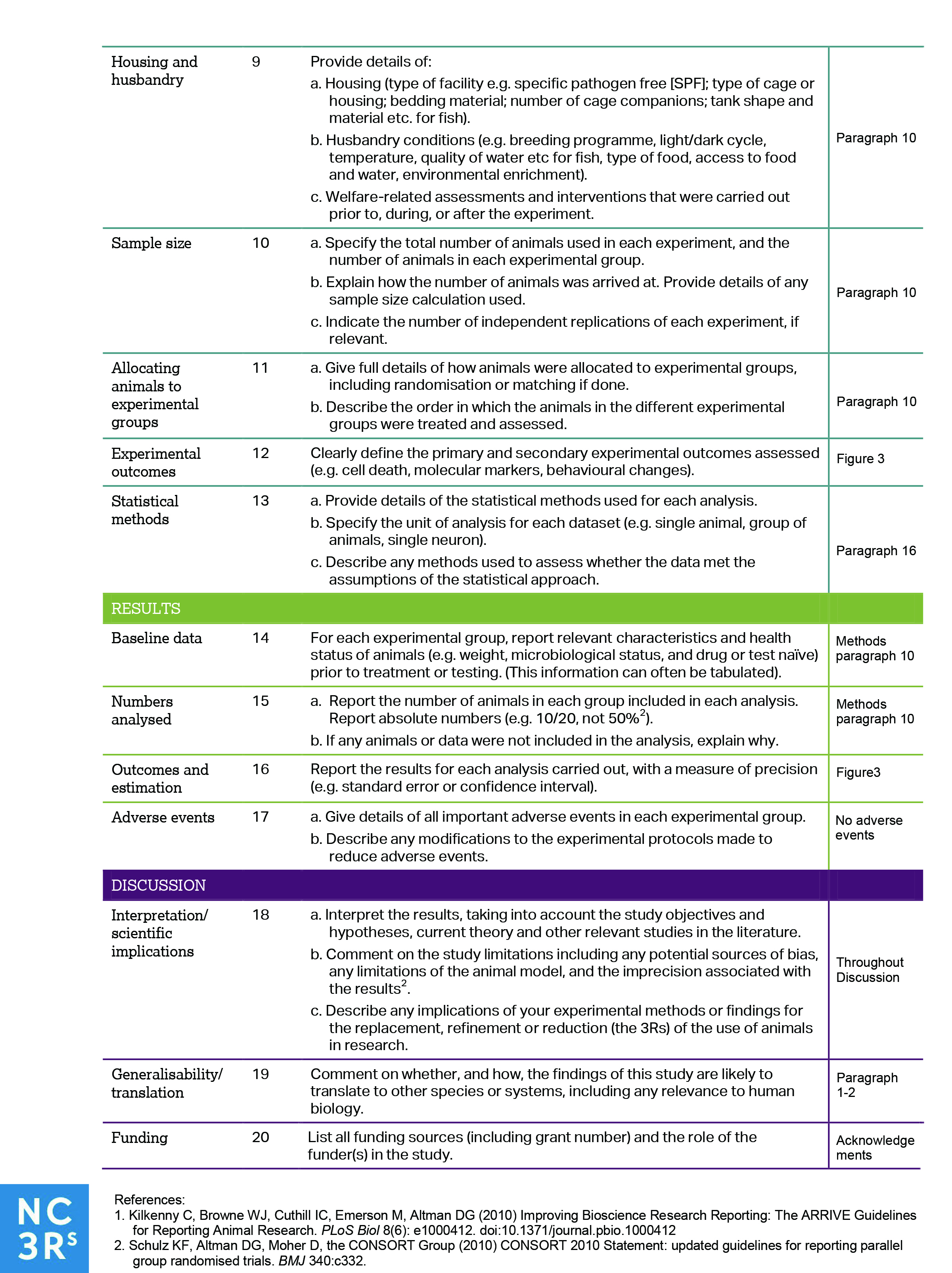


**Ethics Approval and Consent：**


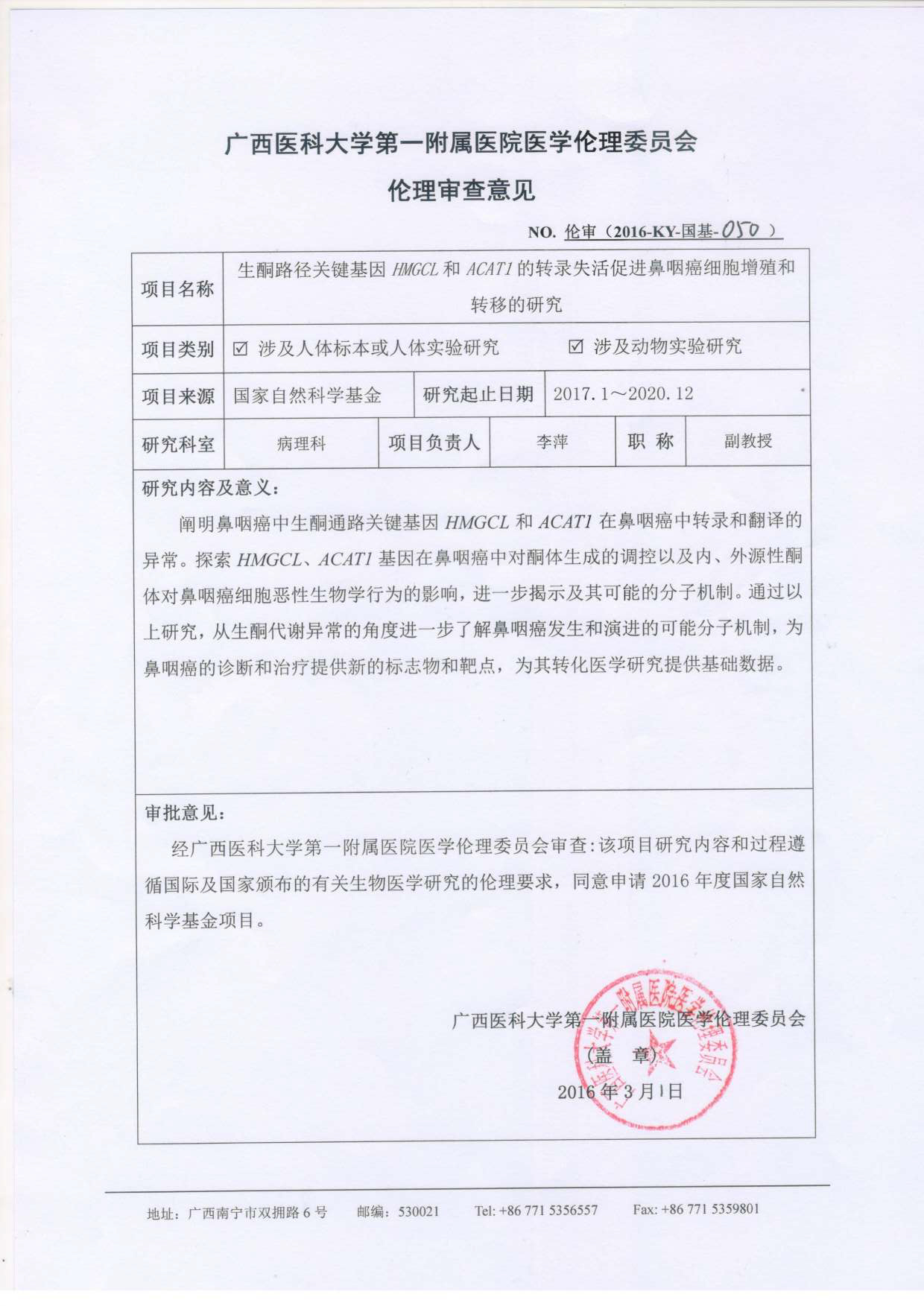


**Cell Line Authentication1：**


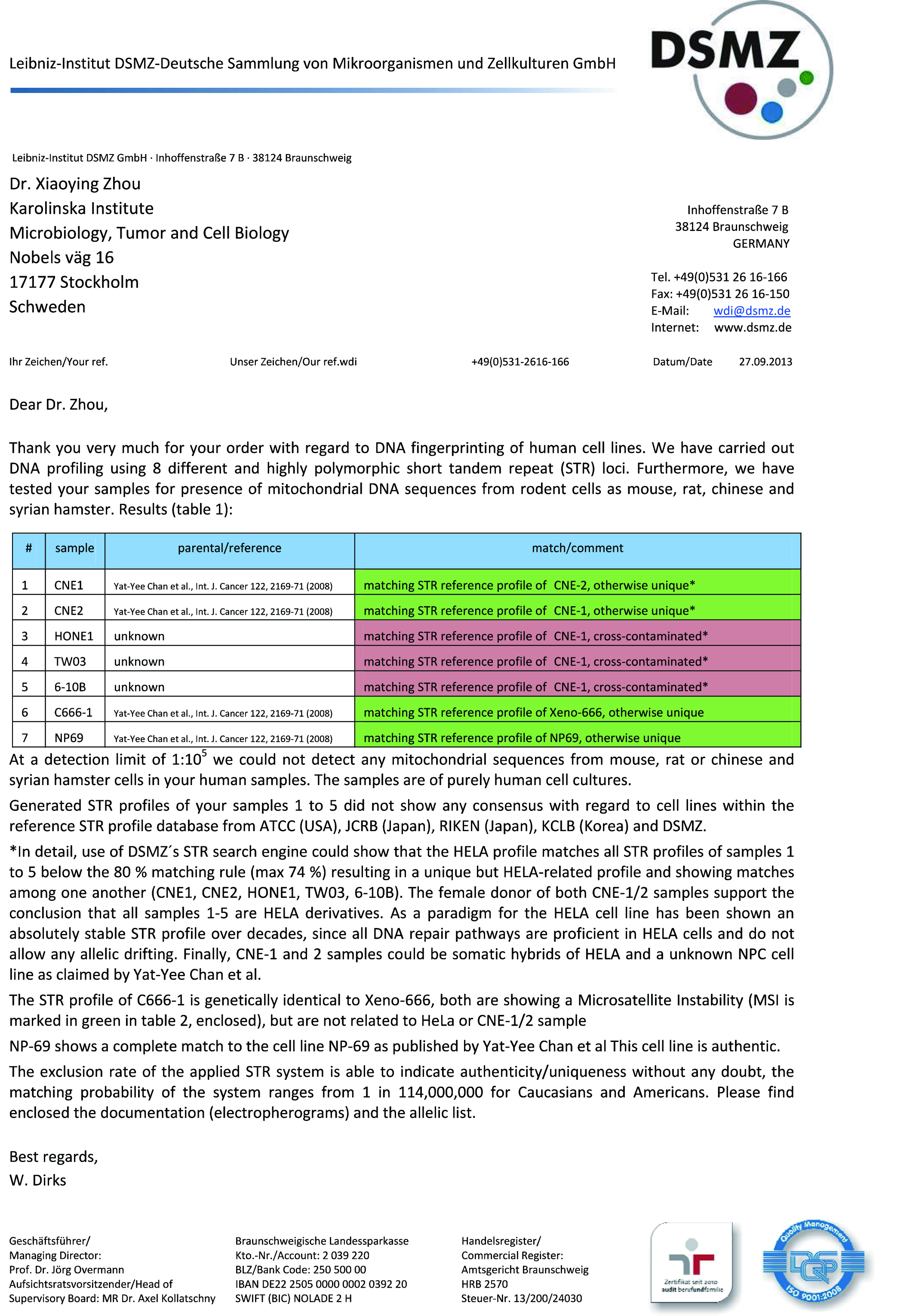


**Cell Line Authentication2：**


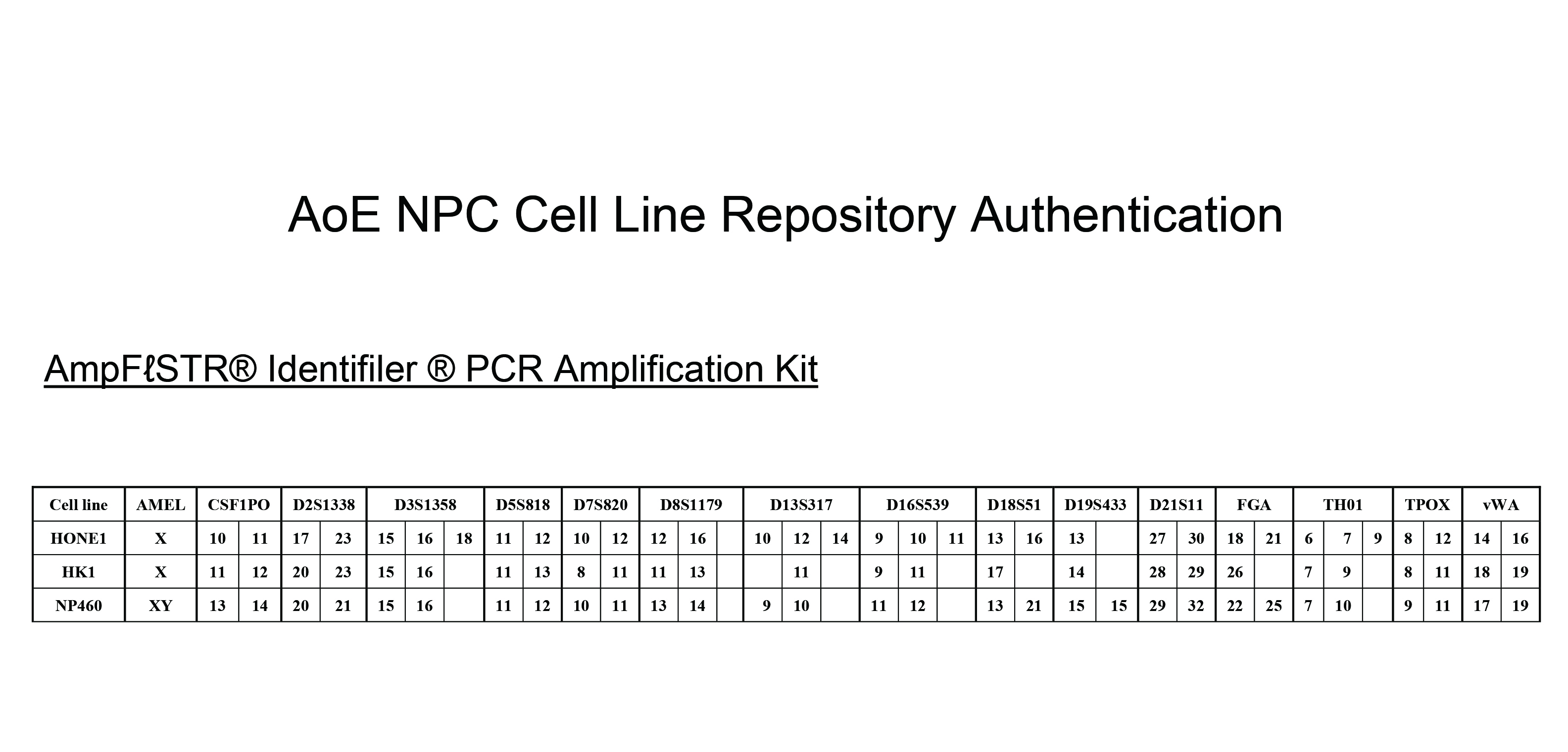


**English-language certification：**


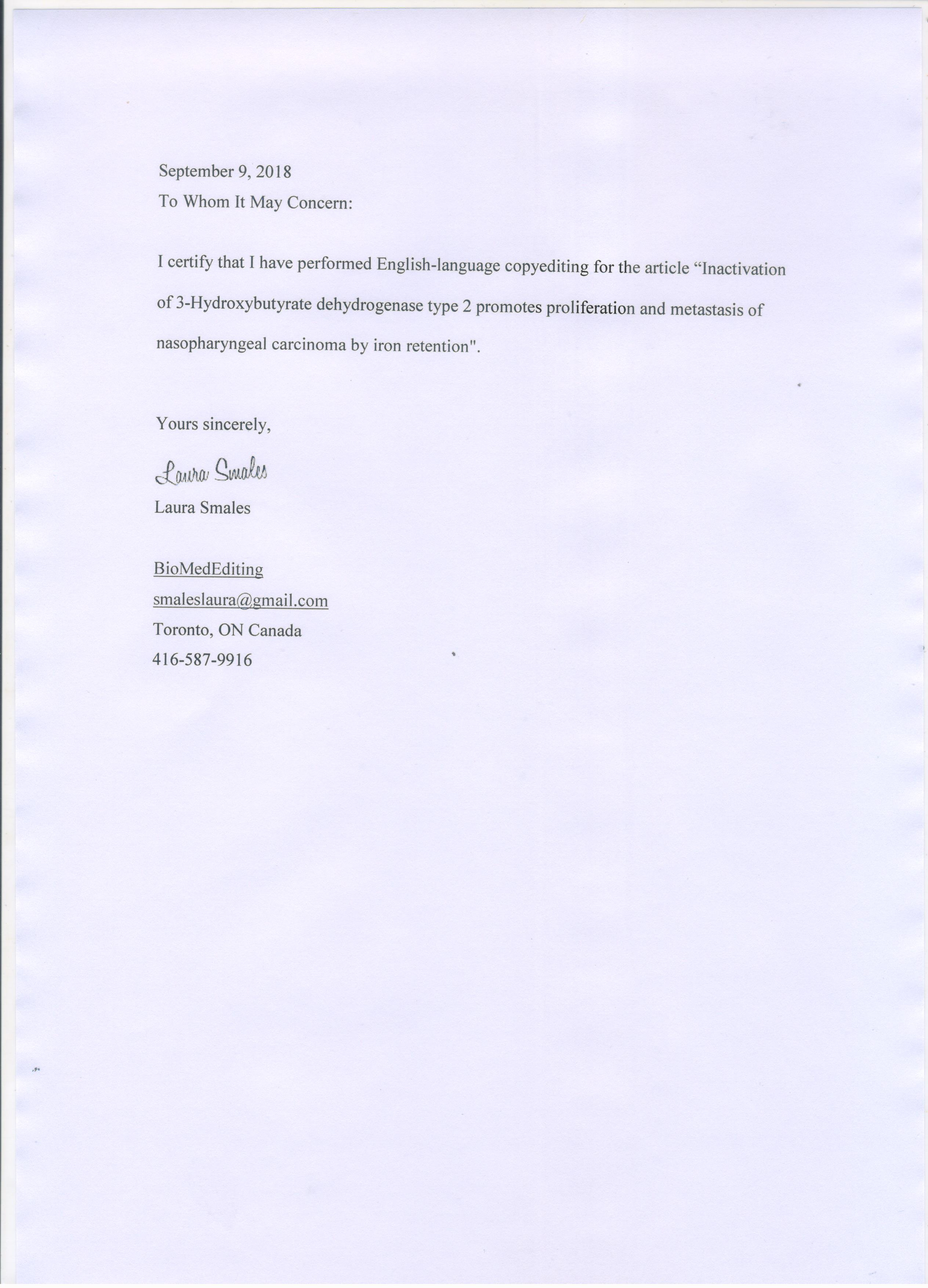

Supplement: Supplementary file 1 — Supplementary one file [file 41416_2019_638_MOESM1_ESM.docx]
